# La trama de una relación compleja: ciencia y catolicismo en Argentina

**DOI:** 10.1590/S0104-59702024000100016

**Published:** 2024-04-15

**Authors:** Esteban Greif

**Affiliations:** i Investigador asistente, Consejo Nacional de Investigaciones Científicas; profesor ayudante, Universidad de Buenos Aires. Buenos Aires – Argentina estebangreif@conicet.gov.ar; estebangreif@conicet.gov.arestebangreif@conicet.gov.ar

Desde el siglo XVIII, la ciencia y la religión han interactuado de diferentes maneras en el territorio de la República Argentina. El estudio de dicha interacción constituye la obra de Miguel de Asúa. Con suma claridad expositiva, dicho estudio considera las relaciones entre los protagonistas políticos y religiosos, así como los grandes movimientos de ideas y los discursos científicos e ideológicos que intervinieron en estas dos grandes áreas de la cultura humana y configuraron las formas particulares de su interacción a lo largo del tiempo.

Su libro es, por varias razones, sumamente revelador y pertinente. En primer lugar, porque comienza a saldar la deuda de un campo de estudios – el de las relaciones entre ciencia y religión en Latinoamérica – que ha sido poco atendido por los especialistas de esta área. En segundo lugar, porque cuestiona y erosiona la lectura, corriente en la historiografía, que entiende el crecimiento del conocimiento científico como la causa del retroceso de la creencia religiosa. Producto de las grandes narrativas de la secularización, dicha lectura no resulta aplicable a la historia argentina. En efecto, tal y como demuestra el autor, fue la dinámica “del proceso de secularización la que contribuyó a modelar las relaciones entre ciencia y religión” (Asúa, 2022, p.1), y no el avance de la primera sobre la última.

En este sentido, Asúa señala desde el comienzo la importancia que posee la “tesis del conflicto” entre ciencia y religión, construcción historiográfica que se nutre de una creencia arraigada en la sociedad que considera estos dos campos de la cultura humana como esencialmente antagónicos. El caso argentino demuestra, como explica Asúa, las raíces ideológicas de esta visión y su funcionalidad en la agenda política de diferentes facciones secularistas, donde los “sucesivos modos de relaciones entre ciencia y religión fueron en gran medida predicadas sobre la dinámica de la secularización” (Asúa, 2022, p.8). La ciencia, por lo tanto, aparecería como un elemento más de la retórica secularista o, para la Argentina, del laicismo asociado a la no creencia y la exclusión de la Iglesia católica de la esfera del gobierno y las instituciones públicas.

En efecto, para el autor, existe otra serie de nociones que mejor expresan las interacciones que se desarrollaron entre ciencia y religión en el territorio nacional: armonía, conflicto o indiferencia ilustran diversos momentos de esta historia que no siempre fue de antagonismo. Como acuerdan sociólogos, historiadores y filósofos de la ciencia y del cristianismo no es el contenido teórico de las teorías científicas sino la visión científica del mundo – o las ideologías cientificistas – las que jugaron un papel central en la secularización del mundo occidental. El caso histórico argentino corrobora esta última afirmación.

El primer capítulo del libro se ocupa del desarrollo científico desplegado por los miembros de la Compañía de Jesús en el territorio de las misiones de Paraguay y el Río de la Plata: la medicina, la historia natural, la astronomía y la cartografía florecieron en un entorno propicio, donde la ciencia habría estado al servicio del trabajo de los misioneros de la Compañía (Asúa, 2014).

En el capítulo dos, Asúa analiza las relaciones entre ciencia y religión desde el fin del período colonial hasta los comienzos de la era independentista en el territorio del Río de la Plata (1770-1820) mientras que, en el tercero, se ocupa de dicha relación fundamentalmente en el territorio de Buenos Aires. El cuarto capítulo aborda el vínculo entre ciencia y religión en el territorio argentino alrededor de 1880, momento en el que la “organización nacional” de Argentina ha concluido en su mayor parte bajo el gobierno de Julio Argentino Roca. El foco está puesto en el impacto de la tesis del conflicto – a partir de la obra de Draper (1874) – y el darwinismo (Levine, Novoa, 2012) en el discurso científico local volcado al servicio de una agenda política secularista.

En el quinto capítulo Asúa presenta el escenario de las relaciones entre ciencia y religión entre finales del siglo XIX y la primera década del siguiente. Desde posiciones ideológicas muy diversas, el proyecto secularista pasó de ser protagonizado por los liberales conservadores a otros actores sociales, como los socialistas, o, incluso, por diversos espiritualismos no cristianos como los teósofos. Frente a este panorama, desde la segunda década del siglo XX comenzó a surgir de la mano de prestigiosos científicos católicos, una defensa de la compatibilidad entre ciencia y catolicismo. En este sentido, Asúa explora (capítulo 6) la carrera científica de una serie de figuras del catolicismo local – Ángel Gallardo y Fortunato Devoto – que desafiaron la visión secularista de la ciencia.

En el capítulo siete, el autor se ocupa de analizar la conceptualización de las relaciones entre ciencia y religión en el período de entreguerras en Argentina, en un momento donde el secularismo se encontraba temporalmente en retirada. En esta sección Asúa examina la visión de diversos científicos católicos sobre la ciencia y la fe en un período de preponderancia del integrismo católico, fuertemente vinculado a la derecha nacionalista de la época. Frente a este grupo de científicos, existió otro, también dentro del catolicismo, pero esta vez comprometidos con la democracia cristiana y posicionamientos políticos más cercanos al liberalismo que, además de ser importantes figuras en el desarrollo científico argentino, desafiaron desde su posición la visión de la ciencia como un patrimonio casi exclusivo del secularismo de liberales y socialistas. De tal manera, Juan Traherne Lewis, Eduardo Braun Menéndez y Augusto Durelli son los protagonistas fundamentales del último capítulo del libro. Asúa analiza su rol protagónico en el período 1932-1959, materializado además en su tarea fundadora de centros científicos privados y la fundación del Instituto Católico de Ciencias (1953-1954) bajo la dirección de Braun Menéndez.

Concluyendo, lo que Asúa logra demostrar en su libro es que, en la historia de las relaciones entre ciencia y religión en Argentina, no fueron las distintas formas como estas se vincularon las que abrieron el camino a la secularización, sino, todo lo contrario, fue la secularización la que determinó como se dieron dichas relaciones.

## References

[B1] ASÚA Miguel de (2022). Science and catholicism in Argentina (1750-1960): a study on scientific culture, religion, and secularization in Latin America.

[B2] ASÚA Miguel de (2014). Science in the vanished arcadia: knowledge of nature in the Jesuit Missions of Paraguay and Río de La Plata.

[B3] DRAPER William (1874). History of the conflict between religion and science.

[B4] LEVINE Alex, NOVOA Adriana (2012). ¡Darwinistas! The construction of evolutionary thought in nineteenth century Argentina.

